# Tracheal intubation with the McGrath MAC X-blade videolaryngoscope in morbidly obese and nonobese patients*

**DOI:** 10.3906/sag-1901-169

**Published:** 2019-10-24

**Authors:** Zehra Ipek ARSLAN, Hadi Ufuk YÖRÜKOĞLU

**Affiliations:** 1 Department of Anesthesiology and Reanimation, Medical Faculty, Kocaeli University, Kocaeli Turkey

**Keywords:** Videolaryngoscope, obese, nonobese, McGrath MAC, X-Blade, intubation

## Abstract

**Background/aim:**

Increased body mass index (BMI) and neck circumference are the two independent predictors of difficult intubation. McGrath MAC X-Blade is a videolaryngoscope specifically designed for difficult intubations.

**Materials and methods:**

Eighty patients with the American Society of Anesthesiologists (ASA) physical status I–III undergoing elective surgery requiring endotracheal intubation were enrolled in the study. Patients were divided into two groups, nonobese (BMI < 30) and morbidly obese (BMI > 35). All patients were intubated with the McGrath MAC X-Blade in both groups. View optimization and tube insertion maneuvers such as reinsertion of the device, slight removal of the device, cricoid pressure, handling force, 90° anticlockwise rotation of the tube, use of stylet, and head flexion maneuvers were recorded. Cormack–Lehane grades, insertion times, intubation, and total intubation times were recorded. The hemodynamic changes and postoperative minor complications were also recorded.

**Results:**

Body mass index, neck circumference, Mallampati scores, and ASA physical status were statistically higher in the morbidly obese group (P < 0.001 and P < 0.05). Sternomental distances were shorter in the morbidly obese (P < 0.05). Cormack–Lehane grades were comparable among the groups. The morbidly obese patients required more reinsertion attempts and cricoid pressure maneuvers during intubation than the nonobese patients (P = 0.019 versus P = 0.012, respectively). Slight removal of the device, handling force, use of the stylet, 90° anticlockwise rotation of the tube, and head flexion maneuvers were also helpful in both groups. Although device insertion times were similar between the groups, intubation and total intubation times were longer in the morbidly obese group (P = 0.009 and P = 0.034, respectively). The groups were comparable in hemodynamic changes and postoperative minor complications.

**Conclusion:**

The McGrath MAC X-Blade videolaryngoscope could safely be used both in nonobese (BMI < 30) and morbidly obese (BMI > 35) patients with the aid of some key maneuvers and with a statistically significant but clinically negligible prolongation of the intubation time.

## 1. Introduction 

The Fourth National Audit Project of the Royal College of Anesthetists and the Difficult Airway Society reported a 4-fold increase in major airway events in obese patients [1]. Dixit et al. [2] demonstrated that Cormack–Lehane 3–4 is higher in morbidly obese patients than in lean patients. Increased body mass index (BMI) and neck circumference are the two independent predictors of difficult intubation [3]. However, most of the difficult airways are unexpected [4]. The anesthetists must minimize multiple attempts at tracheal intubation [5]. The American Society of Anesthesiologists (ASA) guidelines for the management of a difficult airway recommend the use of video laryngoscopes (VLs) in unexpected difficult intubations due to higher frequency of first attempt success rates and improved laryngeal views [6]. Cormack–Lehane views were improved in VL compared to direct laryngoscopy in some trials [7]. If this is the case, VLs must be the first device of choice for every patient with expected difficult intubations or for patients that have one parameter in which difficulty is expected. Therefore, the use of VLs is necessary in obese patients to avoid the use of unnecessary steps [8–10].

In this prospective randomized trial we compared the intubation times and the need of assist maneuvers, hemodynamic changes, and postoperative minor complications between nonobese (BMI < 30) and morbidly obese (BMI > 35) patients intubated with the McGrath MAC X-Blade VL and the slim X-Blade.

Our hypothesis was that the tracheal intubation time of McGrath MAC X-Blade will be prolonged in the morbidly obese (BMI > 35) when compared to nonobese patients (BMI < 30). In addition, morbidly obese patients will require more optimization maneuvers during intubation than lean patients.

## 2. Materials and methods

This study was approved by the Kocaeli Interventional Research Ethics Committee (KİA 2018/584, date 25.12.18) and written informed consent was obtained from all patients. Additionally, this trial was registered to www.ClinicalTrials.com (NCT03759990). After written informed consent was obtained from each patient, 80 patients were enrolled in this prospective randomized study. All patients had an ASA physical status I–III, were between 18 and 70 years of age, and undergoing elective surgery requiring orotracheal intubation. Patients with a history of pregnancy, difficult intubation, with a limited mouth opening <3 cm, full stomach, or upper respiratory tract infection were excluded from this study. Patients were premedicated with midazolam 0.03 mg.kg–1 intravenously in the preoperative care unit. When patients arrived in the operating room, standard anesthesia monitoring was applied, including three channel electrocardiography, noninvasive blood pressure, heart rate, pulse oximetry (SpO2), and end-tidal carbon dioxide. All patients were preoxygenated using a facemask using 5–10 L min–1 100% O2 for 3–5 min in the supine position. Demographic (age, sex, weight, height, BMI, ASA physical status) and airway variables (thyromental distance, sternomental distance, interincisor distance, neck circumference, Mallampati, head extension, mandibular protrusions [A, lower incisors protruded more than upper incisors; B, lower incisors could be brought edge to edge with the upper incisors; C, lower incisors could not be brought to the upper incisors]), and teeth morphology (full/lack/absent) were recorded. All patients were intubated with the McGrath MAC using X-Blade size 3 that was inserted like the Macintosh laryngoscope (Figure). Patients were divided into lean (BMI < 30) and obese (BMI > 35) groups using a sealed envelope technique. General anesthesia was induced with intravenous (iv) propofol 3 mg kg–1 and fentanyl 1 mg kg–1 (calculated according to lean body weight with the 24 × [height2] formula). Ease of facemask ventilations were recorded according to the following categorization; easy, airway, two-person, oxygen flush, and impossible [11]. Then, 0.6 mg kg–1 iv rocuronium was administered for muscle relaxation. Patients were manually ventilated until the switch on the acceleromyograph (TOF-Watch SX, Organon, Dublin, Ireland) disappeared. The insertion time, orotracheal intubation time, total orotracheal intubation time, and Cormack–Lehane grades of the patients were recorded. A 7.5 mm polyvinylchloride endotracheal tube was used for women and 8.0 mm for men in both groups. All tubes were lubricated with a 10% lidocaine spray before intubation. All intubations were performed by anesthetists with at least 5 years of anesthesia experience and by individuals that also had at least 50 successful intubations with the McGrath MAC X-Blade. A jelly pillow was placed under each patient’s head and the patient remained in the supine position during the entire intubation process in both groups.

**Figure:**
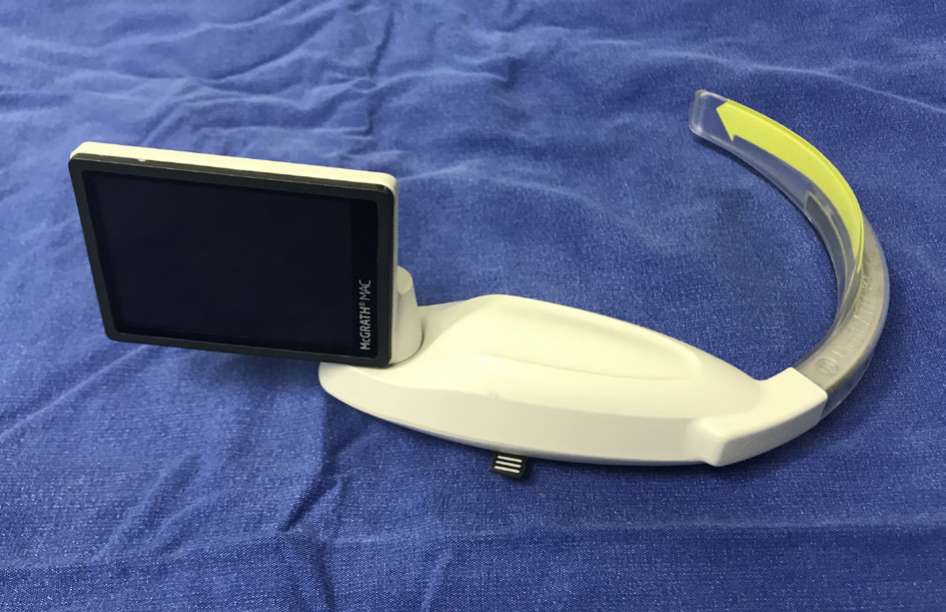
McGrath MAC and slim X-Blade.

## 2.1. Insertion time

Defined as the time that elapsed from the moment the device entered the oral cavity until optimal glottic visualization was achieved. Reinsertion of the device, handling force, the need for another attempt maneuvers were included in this time period.

## 2.2. Orotracheal intubation time

Defined as the time that elapsed from the moment the device entered the oral cavity until the visualization of the tube entering through the vocal cords. If resistance was encountered during tube adjustment then assist maneuvers were applied to direct the tube into the trachea, which included the slight removal of the device to the mouth, cricoid pressure, 90° anticlockwise rotation maneuver of the tube, head flexion maneuvers, and the need for inserting a stylet into the tube. If the tube was felt to be posterior, first we applied a slight removal of the device maneuver, then cricoid pressure and the head flexion maneuver, and stylet insertion into the endotracheal tube in random order. If a standard Mallinckrodt stylet (Mallinkrodt Inc., Blanchardtown, Dublin, Ireland) was needed, it was shaped like a “hockey-stick”, then the stylet inserted into endotracheal tube was advanced from the right corner of the mouth. As soon as the endotracheal tube was advanced to the glottis, the intubating stylet was withdrawn and the endotracheal tube advanced into the trachea. If the endotracheal tube was felt to be positioned laterally, then a 90° anticlockwise rotation maneuver was applied.

## 2.3. Total orotracheal intubation time

Defined as the time that elapsed from the moment the device entered the oral cavity until the confirmation of intubation from the capnograph.

Anesthesia was maintained with 2% sevoflurane in a mixture of nitrous oxide and oxygen. Systolic blood pressure, diastolic blood pressure, mean arterial pressure (MAP), heart rate (HR), and SpO2 were recorded at baseline (preoperatively), after anesthesia induction, after insertion of the device, just after intubation, and at 2 min intervals after intubation for 5 min by an independent unbiased observer. If the patient could not be intubated after three attempts or after 120 s, it was recorded as a failed intubation. SpO2 < 92 was recorded as hypoxemia. Esophageal intubation, teeth, tongue, mouth, or mucosal damage (bloodstaining on the device) were also recorded. Minor complications such as sore throat, hoarseness, dysphagia, bronchospasm, nausea and vomiting were recorded by a blinded unbiased observer at the postoperative care unit. Tramadol 1 mg kg–1, paracetamol 10 mg kg–1, and ondansetron 4 mg kg–1 were administered intravenously to prevent postoperative pain and vomiting.

We based our sample size according to Yumul et al. [12]. They used the McGrath MAC in morbidly obese patients and they found the total intubation time to be 62 ± 31 s. Lee et al. [13] used the McGrath MAC in normal patients and found the total intubation time of nonobese patients to be 32 (27–35) s. Based on this data, for a = 0.05 and b = 0.2 we calculated our sample size as 40 patients in each group. Continuous data were examined for normal distribution with the Kolmogorov–Smirnov test. We used Student’s t-test for normally distributed data and the Mann–Whitney U test for nonnormally distributed data. Normally distributed data were given as mean ± standard deviation (SD) and nonnormally distributed data as median (25–75 percentiles). Categorical data were calculated with the chi-squared test. P < 0.05 was considered to be statistically significant. 

## 3. Results 

Height, weight, BMI, sternomental distances, neck circumference, Mallampati score, and ASA physical status were statistically different between the groups (Table 1). Groups were comparable in age, thyromental distance, interincisor distance, sex, and tooth morphology (Table 1). All patient mandibular protrusions were classified as A and head flexion and extension capacities were normal. Mask ventilations were successful and statistically similar in both groups (Table 2). All nonobese and morbidly obese patients were intubated successfully, and intubation occurred on the second attempt at the most. Cormack–Lehane grades were comparable among the groups. Morbidly obese patients required more reinsertion and cricoid pressure maneuvers during intubation than nonobese patients (P = 0.019 versus P = 0.012) (Table 2). Slight removal of the device, handling force, use of stylet, 90° anticlockwise rotation, and head flexion maneuvers were also helpful in both groups (Table 2). Although device insertion times were similar between the groups, intubation and total intubation times were longer in the morbidly obese group (P = 0.009 versus P = 0.034, respectively) (Table 3). Mucosal damage occurred in 1 patient in the nonobese group. No teeth, mouth, or tongue damage, or esophageal intubation was detected in any patient in any group. Groups were comparable regarding postoperative minor complications such as sore throat and dysphagia (Table 3). There were no statistically significant differences detected in the HR and MAP changes during the procedure. Postoperative hoarseness was detected in 1 patient in the morbidly obese group. No bronchospasm or hypoxia was detected in any of the patients.

**Table 1:** Demographic variables and airway characteristics of patients. Values are given as mean ± SD and median [25–75 percentiles] or as numbers.

	Nonobese McGrath MAC X-Blade (N = 40)	Morbidly obese McGrath MAC X-Blade (N = 40)	P
Age; years	54.00 [38.25–62.50]	50.50 [46.00–61.50]	0.714
Height; cm	168.10 ± 9.65	161.70 ± 7.31	0.001*
Weight; kg	74.80 ± 11.18	107.70 ± 16.99	<0.001^&^
BMI; kg m^–2^	26.72 [24.26–28.63]	40.25 [37.46–44.72]	<0.001^&^
Thyromental distance; cm	8.00 [7.00–8.00]	7.00 [7.00–8.00]	0.208
Sternomental distance; cm	15.00 [14.00–16.00]	14.00 [12.00–15.00]	0.007*
Neck circumference; cm	40.00 [36.25–41.00]	43.00 [41.00–45.00]	<0.001^&^
Interincisor distance; cm	4.25 [4.00–4.88]	4.00 [4.00–5.00]	0.557
ASA; 1 / 2 / 3	23 / 16 / 1	7 / 31 / 2	0.001*
Sex; F / M	18 / 22	25 / 15	0.116
Mallampati; 1 / 2 / 3	27 / 13 / 0	16 / 21 / 3	0.021*
Teeth morphology; full / lack / absent	33 / 4 / 3	32 / 5 / 3	0.851

**Table 2:** Airway management variables of patients. Values are given as numbers.

	Nonobese McGrathMAC X-Blade (N = 40)	Morbidly obese McGrath MAC X-Blade (N = 40)	P
Mask ventilation; easy / airway / two handed	18 / 17 / 5	8 / 23 / 9	0.053
Cormack–Lehane; 1 / 2	29 / 11	29 / 11	1.000
Number of intubation attempts; 1 / 2	33 / 7	30 / 10	0.412
Cricoid pressure; present / absent	10 / 30	21 / 19	0.012*
Handling force; present / absent	4 / 36	8 / 32	0.210
Reinsertion maneuver; present / absent	21 / 19	31 / 9	0.019*
90° anticlockwise rotation maneuver; present / absent	6 / 34	6 / 34	1.000
Head flexion maneuver; present / absent	3 / 37	5 / 35	0.456
Slight removal of the device maneuver; present / absent	9 / 31	9 / 31	1.000
Stylet use; present / absent	4 / 36	4 / 36	1.000
Maneuver; present / absent	26 / 14	34 / 6	0.039*

*P < 0.05; ^&^P < 0.001

**Table 3:** Airway management variables. Values are given as median [25–75 percentiles] or as numbers.

	Nonobese McGrath MAC X-Blade (N = 40)	Morbidly obese McGrath MAC X-Blade (N = 40)	P
Insertion time; s	4.00 [3.63–5.00]	4.00 [4.00–6.00]	0.445
Intubation time; s	13.00 [10.00–18.00]	18.00 [13.00–28.00]	0.009*
Total intubation time; s	25.00 [22.00–30.00]	31.00 [21.25–41.75]	0.034*
Postoperative sore throat; present / absent	6 / 34	3 / 37	0.288
Postoperative dysphagia; present / absent	4 / 36	3 / 37	0.692

*P < 0.05

## 4. Discussion 

The main result of this study was that the prolongation of tracheal intubation with the McGrath MAC X-Blade in morbidly obese patients compared to nonobese patients is statistically significant but clinically negligible. We could use especially cricoid pressure and reinsertion maneuvers to improve our success while inserting the tube into the trachea.

Difficult intubation and desaturation rates were higher (15.5% versus 2.2%) in morbidly obese patients than in nonobese patients [14]. Obesity is associated with a higher risk of adverse events and a lower first intubation success rate [15].

VLs were found to be superior to the Macintosh laryngoscope for tracheal intubation in adults with obesity [16,17]. VLs improve the laryngeal view, increase the first attempt success rate, and reduce patient trauma. Based upon these observations, direct laryngoscopy should be abandoned altogether and VLs should become the first line approach for intubation for a patient with an expected difficult airway. The experience of the provider is an essential factor that must not be underestimated [18].

In the Lee et al. study [2], various types of providers presented results demonstrating that these differences in tracheal intubation times were too long when compared to our results. In contrast with our results, tracheal intubation was performed in <20 s with the McGrath MAC in a patient with cervical immobilization [19]. Morbidly obese patients were intubated with the McGrath MAC in a shorter time duration than the other VLs [12]. This was due to X-Blade’s slim design.

A recently published trial demonstrated that the Cormack–Lehane grade was lower and tracheal intubation time was approximately 46 s in the McGrath MAC compared to the Macintosh laryngoscope. Esophageal intubation with the McGrath MAC was lower in expert hands and postoperative minor complications were similar among the groups [20]. The McGrath MAC was superior to other VLs in experienced hands in terms of the first intubation success rate and the duration of tracheal intubation and hypoxemia rate. Equivalent to our results, the median time for successful intubation with the McGrath MAC was 17 s in normal airways. Groups were comparable in laryngeal views, number of intubation attempts, need for assist maneuvers, and airway trauma. Operators with different levels and types of skill experience (attending, anesthesia resident, anesthesia technician, student nurse anesthetist) were present in this trial [21].

A recent letter to the editor showed that the McGrath MAC is also a good intubation device providing a good view in super obese patients [22]. There is a published case report of a severely obese patient with a large goiter that was successfully intubated with the McGrath MAC [23].

The average tracheal intubation time was longer in most obese patients during intubation with VLs. Inserting the VL is easier in nonobese patients than in the morbidly obese [24,25]. In our study we inserted the McGrath MAC X-Blade for similar duration times both in nonobese and morbidly obese patients.

We used the McGrath MAC as an indirect laryngoscope by experts. The direct laryngoscope view of the McGrath MAC was poorer than its indirect view in both novice and expert hands. The stylet and cricoid pressure maneuver were used to facilitate their intubation process [26].

Cricoid pressure also improves the Cormack–Lehane grade of the McGrath MAC. In this trial, as with our study, the McGrath MAC provides equal intubation times (approximately 13 s) in nonobese patients with the Macintosh and minimal hemodynamic changes compared to other VLs with the help of some key maneuvers [27]. We also used the cricoid pressure maneuver to facilitate tube insertion through the vocal cords. Even though obese patients needed more reinsertion and cricoid pressure maneuvers, there was no statistically significant difference detected according to the occurrence of postoperative sore throat between the groups.

Gaszynski published a case series of super obese patients who were intubated with the McGrath MAC, and the Cormack–Lehane grades were better than the other VLs in these patients [26]. Cormack–Lehane grades of nonobese and morbidly obese patients were similar with the McGrath MAC in our study.

Obtaining a good view of the vocal cords does not guarantee intubation. This is a rule for all types of VLs [8]. As with the previous trials, we needed to use a stylet [4], reinsertion and withdrawal of the blade [28], and a 90° anticlockwise rotation of the tube maneuvers [29] to increase the intubation success rate. Use of a stylet and applying the drawback (slight removal of the device to the mouth) maneuver was helpful for an endotracheal tube that had fallen posteriorly. Some authors additionally changed the tube to a larger one to overcome a posteriorly falling endotracheal tube [8], and the 90° anticlockwise rotation of the tube is necessary for a laterally fallen endotracheal tube. However, these are not the complete solutions in our study as well. The head flexion maneuver was effective in some patients during nasotracheal intubation and directing the tube into the trachea with the McGrath MAC [30].

The McGrath MAC caused less hemodynamic changes during tracheal intubation compared to direct laryngoscopy and other VLs [31].

There are some limitations to our study. First, our sample size was calculated according to the intubation times not for the minor postoperative complications and not for the maneuvers. Second, our study was performed by experienced users of the McGrath MAC; the results would be different if this study was performed by inexperienced users. Third, we put jelly pillows under each patient’s head; if we had not, the result would have been different. Fourth, we used only size 3 X-Blade for nonobese and morbidly obese patients, but if different sizes were available then the results would have been different too.

In conclusion, the McGrath MAC X-Blade video laryngoscope could safely be used both in nonobese (BMI < 30) and morbidly obese (BMI > 35) patients with the help of some key maneuvers.
